# Bacterial cell membranes and their role in daptomycin resistance: A review

**DOI:** 10.3389/fmolb.2022.1035574

**Published:** 2022-11-14

**Authors:** April H. Nguyen, Kara S. Hood, Eugenia Mileykovskaya, William R. Miller, Truc T. Tran

**Affiliations:** ^1^ Center for Infectious Diseases Research, Houston Methodist Research Institute, Houston, TX, United States; ^2^ Division of Infectious Diseases, Department of Medicine, Houston Methodist Hospital, Houston, TX, United States; ^3^ Department of Biochemistry and Molecular Biology, McGovern Medical School, University of Texas Health Science Center, Houston, TX, United States

**Keywords:** daptomycin, resistance, phospholipids, enterococci, *Staphylococcus aureus*, streptococci

## Abstract

Lipids play a major role in bacterial cells. Foremost, lipids are the primary constituents of the cell membrane bilayer, providing structure and separating the cell from the surrounding environment. This makes the lipid bilayer a prime target for antimicrobial peptides and membrane-acting antibiotics such as daptomycin. In response, bacteria have evolved mechanisms by which the membrane can be adapted to resist attack by these antimicrobial compounds. In this review, we focus on the membrane phospholipid changes associated with daptomycin resistance in enterococci, *Staphylococcus aureus*, and the Viridans group streptococci.

## Introduction

The cell membrane is a vital component of the bacterial cell, serving as a part of the protective barrier against the surrounding environment and as a scaffold for metabolic and regulatory proteins. Bacterial membranes are primarily composed of a bilayer of phospholipids with varying headgroups, acyl chain lengths, and acyl saturation which can influence membrane properties such as fluidity or charge. The major lipids in Gram-positive bacteria, particularly the firmicutes, are anionic phospholipids (APLs) [e.g., phosphatidylglycerol (PG), cardiolipin (CL)] and their derivatives (lysyl-PG, alanyl-PG), zwitterionic phospholipids, as well as other lipid classes like glycolipids and diacylglycerols (Sohlenkamp and Geiger, 2016). While the traditional “fluid mosaic model” describes a uniform bilayer whereby lipids and proteins are free to diffuse throughout the space, there is increasing evidence of the existence of distinct domains within the membrane (Matsumoto et al., 2006). These phospholipid domains have been described across several clinically important species of Gram-positive organisms, including “functional membrane microdomains” in *S. aureus* (García-Fernández et al., 2017), APL microdomains in *Enterococcus faecalis* (Tran et al., 2013a)*,* or the ExPortal of *Streptococcus* spp. (Vega and Caparon, 2013). Importantly, alterations in these domains have been associated with specific roles in the bacterial response to antibiotics and antimicrobial peptides active at the cell envelope.

The relative accessibility and essential functions of the bacterial membrane components make them effective targets for antimicrobials. The rise of multidrug resistant organisms such as methicillin-resistant *Staphylococcus aureus* (MRSA) and vancomycin-resistant enterococci (VRE) (Munita et al., 2015; Khan et al., 2018) spurred interest in antibiotics with alternative mechanisms of action that could bypass resistance to available agents. Daptomycin (DAP) has emerged as a treatment option that retained *in vitro* activity against resistant Gram-positive organisms and has seen increasing use against invasive infections due to MRSA and particularly VRE (Jorgensen et al., 2003; Mortin et al., 2007). Resistance to DAP is being reported with increasing frequency in clinical isolates, approaching 15%–28% in centers with heavy use of DAP (Kamboj et al., 2011; Munita et al., 2015; DiPippo et al., 2017; Wang et al., 2018). This review will address the role of the cell membrane in DAP resistance (DAP-R), with a focus on the alteration and adaptation of membrane phospholipids in Gram-positive bacteria of clinical importance.

## Mechanism of action of daptomycin

DAP is a lipopeptide antibiotic originally isolated from *Streptomyces roseosporus* in the 1980s (Eisenstein et al., 2010). DAP possesses a cyclic peptide core linked to a fatty acyl chain and requires calcium to adopt an amphipathic conformation that facilitates oligomerization and insertion into the bacterial membrane. In addition, complex formation with calcium masks the lipopeptides’s negative charge and increases its affinity for the membrane lipid PG, the major constituent of Gram-positive membranes (typically comprising 50%–65% of the total APL content in *S. aureus* and *E. faecalis* (Müller et al., 2016; DeMars et al., 2020; Kotsogianni et al., 2021; Woodall et al., 2021). Recent structural analysis of Ca^2+^-DAP in lipid bilayers containing PG shows that DAP forms tetramers within the outer leaflet (Beriashvili et al., 2020). These tetramers can reversibly flip between the outer and inner leaflets and associate with one another to form a complex that spans the entire membrane (Zhang et al., 2014), resulting in membrane leakage and depolarization in cells after prolonged incubation with Ca^2+^-DAP (Silverman et al., 2003).

**FIGURE 1:**
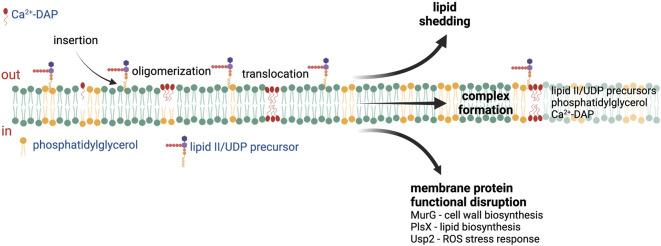
Proposed mechanisms of action of daptomycin. After insertion into the cell membrane, the Ca^2+^-daptomycin complex oligomerize in the outer leaflet of the cell membrane. Ca^2+^-daptomycin oligomers are translocated to the inner leaflet of the cell membrane which result in lipid shedding, tripartite formation with phosphatidylglycerol and lipid II/UDP precursors, or membrane protein functional disruption. See text for details. ROS—reactive oxygen species, UDP—undecaprenyl. Figure produced with BioRender.

Multiple DAP mechanisms of action have been proposed based on the different reported cellular responses to DAP exposure (Figure 1), including membrane permeabilization, inhibition of cell wall synthesis, and physical alteration of membrane fluidity or curvature (Mengin-Lecreulx et al., 1990; Silverman et al., 2003; Pogliano et al., 2012; Müller et al., 2016). A recent study published by Grein et al. demonstrated that in *S. aureus* Ca2^+^-DAP oligomers form a tripartite complex with PG and lipid II (or other undecaprenyl cell envelope precursors, UDP), which are primarily located at the cell septum (Figure 1). The DAP-PG-lipid II complex not only sequesters lipid II substrate, but also disrupts the localization and assembly of the peptidoglycan synthesis machinery (Grein et al., 2020). In *Bacillus subtilis*, the insertion of DAP complexes in the membrane and subsequent alteration in membrane fluidity leads to mislocalization of crucial membrane proteins (Müller et al., 2016). After prolonged incubation, the DAP-UDP-PG complexes spread throughout the membrane, compromising envelope integrity and leading to cell death (Grein et al., 2020). The ability of DAP to disrupt cell wall biosynthesis is also supported by multiple *in vitro* and *ex vivo* studies where synergy or re-sensitization of DAP was observed upon combination with cell-wall acting antibiotics such as β-lactams (Dhand et al., 2011; Sakoulas et al., 2013; Sakoulas et al., 2014; Smith et al., 2015a; Smith et al., 2015b; Werth et al., 2015; Yim et al., 2017; Kebriaei et al., 2019; Johnson et al., 2021). Additionally, DAP has been shown to induce production of reactive oxygen species in *S. aureus* via binding to the universal stress response protein Usp2, a membrane protein which has been postulated to mediate the response to oxidative stress (Po et al., 2021). Thus, the binding and action of DAP is tied to the specific properties of the target bacterial membrane.

## Alterations of membrane lipids associated with changes in daptomycin susceptibility

A recurring theme in the emergence of DAP resistance across species is the presence of mutations leading to changes in both proteins of the lipid metabolic pathways and two-component sensors (TCS) involved in regulating cell envelope homeostasis (Tran et al., 2015). These changes can lead to alterations in the synthesis and modification of membrane lipid species and acyl-groups, thus impacting the composition and properties of the membrane (Table 1). In addition to *de novo* synthesis, bacteria can utilize exogenous fatty acids from their environment, including from human hosts, and importantly these exogenous acyl-chain profiles may be substantially different from those achievable by *de novo* synthesis in bacteria (Parsons et al., 2014a; Parsons et al., 2014b; Saito et al., 2014). These findings are also seen in model membranes where phospholipid head group and fatty acid residues in phospholipids can influence the membrane properties and alter the sensitivity to DAP (Beriashvili et al., 2018). The specific mechanisms by which these alterations impact the membrane are the subject of ongoing investigation, but shifts in membrane lipid content, fatty acid saturation, and alteration of membrane surface charge are common features of resistant isolates (Table 1).

**TABLE 1:** Genes associated with membrane changes and daptomycin resistance.

Organism	Relevant gene	Predicted function	Phospholipids and fatty acids impacted	Surface charge and fluidity/rigidity	Proposed mechanism of resistance	References
*Enterococcus faecalis*	*liaF*	three-component regulatory system	• alterations in PG	• alterations in surface charge	APL microdomain redistribution	Arias et al. (2011); Miller et al. (2019)
	*cls*	cardiolipin synthase	• alteration in diglycodiacylglycerol			
	*gdpD*	glycerophosphodiester phosphodiesterase	• alteration in L-PGalteration in CL			
	*dak*	fatty acid kinase				
*Enterococcus faecium*	*cls*	cardiolipin synthase	• alterations in PG	• alterations in surface charge	Increased net surface charge	Tran et al. (2013b); Diaz et al. (2014); Prater et al. (2019)
	*cfa*	cyclopropan-fatty-acyl-phospholipid synthase	• alterations in L-PG	• alterations in membrane rigidity/fluidity	APL microdomain redistribution	
	*dlt*	D-alanylation of techoic acid	• alteration in digalactosyldiacylglycerols			
	*mprF*	multiple peptide resistance factor	• alterations in unsaturated and cyclic fatty acids			
	*yvcRS*	ABC transporter				
	*oatA*	O-acetyltransferase				
	*divIVA*	cell division and chromosome segregation				
*Staphylococcus aureus*	*vraSR*	two-component stress response system	• alterations in PG	• alterations in surface charge	Increased net surface charge	Peschel et al. (2001); Mishra et al. (2011a); Mehta et al. (2012); Peleg et al. (2012); Mishra and Bayer (2013); Hines et al. (2017); Jiang et al. (2019)
	*yycFG*	two-component cell wall biosynthesis	• alterations in L-PG	• alterations in membrane rigidity/fluidity	Decrease/loss of target phospholipids	
	*pgsA*	CDP-diacylglycerol-glycerol-3-phosphate-3-phosphatidyltransferase	• alternations in CL			
	*cls*	cardiolipin synthase	• no changes in fatty acids			
	*mprF*	multiple peptide resistance factor				
*Viridans groupstreptococci*	*cdsA*	phosphatidate cytidylyltransferase	• alterations in PG	• alterations in surface charge	Decrease/loss of target phospholipids	Akins et al. (2015); Adams et al. (2017); Mishra et al. (2017); Kebriaei et al. (2019); Tran et al. (2019)
			• alterations in PA	• alterations in membrane rigidity/fluidity		
	*pgsA*	CDP-diacylglycerol-glycerol-3-phosphate-3-phosphatidyltransferase	• alternations in CL			

APL, anionic phospholipid; CL, cardiolipin; L-PG, lysyl-phosphatidylglyerol; PA, phosphatidic acid; PG, phosphatidylglycerol

### *Enterococcus faecalis* and *Enterococcus faecium*

**FIGURE 2:**
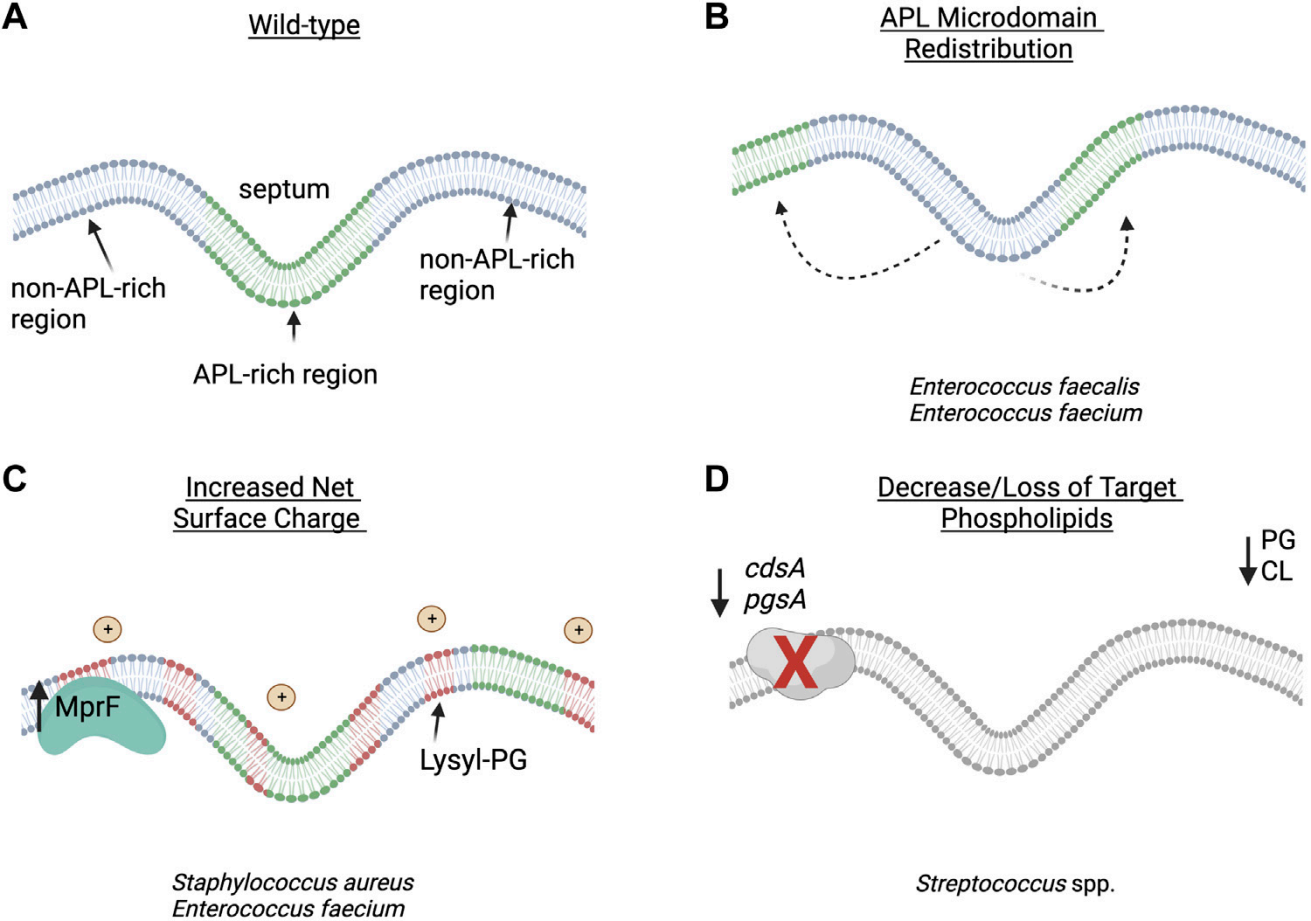
Proposed strategies of daptomycin resistance in Gram-positive pathogens: **(A)** wild-type bacterial cell membrane with anionic phospholipid (APL) microdomains clustered at the division septum. **(B)** In *Enterococcus faecalis*, redistribution of APL diverts daptomycin (DAP) away from the critical division septum. **(C)** In *Staphylococcus aureus* and *E. faecium*, changes in membrane phospholipid content and net surface charge lead to decreased daptomycin binding and oligomerization to the cell membrane. **(D)** In Viridans group streptococci, the decrease or loss of target phospholipid, PG and CL, are associated with overall decreased binding to the cell membrane or hyperaccumulation of daptomycin in a small subset of the bacterial population. APL–anionic phospholipid; CL–cardiolipin; PG–phosphatidylglycerol. Figure produced with BioRender.

*E. faecalis* and *E. faecium* are important opportunistic human pathogens that cause a variety of infections ranging from skin and soft tissue infections to bacteremia and endocarditis (Krawczyk et al., 2021). DAP has emerged as a front-line agent against complicated VRE infections; however, emergence of DAP-R has threatened its utility in clinical practice. Characterization of both lab-evolved and clinical DAP-R strains has shed light on the genetic and phenotypic changes associated with DAP-R in enterococci (Arias et al., 2011; Palmer et al., 2011; Tran et al., 2013b; Miller et al., 2013; Diaz et al., 2014; Wang et al., 2018; Miller et al., 2019). In *E. faecalis*, there is an observable re-distribution of APL microdomains, while in *E. faecium* a DAP repulsion phenotype similar to *S. aureus* is seen (Ernst et al., 2009; Tran et al., 2013a; Diaz et al., 2014; Khan et al., 2019) (Figure 2).

Enterococci predominantly contain the APL PG in their cell membranes, in addition to CL, cationic PG derivatives such as lysyl-PG, and various glycolipids and diacylglycerols. In DAP-R enterococcal isolates, several general patterns in membrane lipid changes begin to emerge. Two studies using the clinical strain pair *E. faecalis* S613 (DAP-S) and R712 (DAP-R) grown to stationary phase found that the DAP-R isolate had a significant reduction in the amount of membrane PG as compared to its DAP-S parent, and an increase in glycerolphospho-diglycodiacylglycerol (GP-DGDAG) (Mishra et al., 2012; Hines et al., 2017). Further, an analysis of the laboratory strain *E. faecalis* OG1RF and two derivative strains rendered DAP-R via *in vitro* passage showed decreases in PG and lysyl-PG in the resistant isolates during mid-logarithmic phase growth (Rashid et al., 2017). In paired isolates of *E. faecium*, the DAP-R isolate showed a significant decrease in PG (14% vs. 33%) and increases in GP-DGDAG (23% vs. 12%) as compared to the susceptible parent strain (Mishra et al., 2012). Taken together, these data appear to indicate that a relative loss of PG, the primary phospholipid involved in DAP binding, may contribute to the resistant phenotype. Interestingly, a different analysis of membrane lipids of the DAP-R *E. faecalis* strain performed by 2D-TLC during exponential growth showed a significant increase in PG, a reduction in CL, and no differences in lysyl-PG as compared to S613, while levels of GP-DGDAG were not reported (Khan et al., 2019). Thus, lipid alterations other than a decrease in membrane PG are likely to also contribute to DAP-R. A recent study showed that the absence of CL in *E. faecalis* OG1RF, achieved by deletion of both CL synthases, increased sensitivity to DAP and other cell damaging agents such as SDS, while the absence of lysyl-PG, resulting from deletion of *mprF2*, did not change sensitivity to DAP but rendered the mutant more resistant to SDS (Woodall et al., 2021). The triple mutant, which lacked CL and lysyl-PG, restored membrane tolerance to DAP and SDS to the level of parental strain OG1RF. These results further demonstrate the complexity of membrane lipid adaptation to DAP and other cell membrane damaging compounds.

The importance of cell surface charge in the DAP-R phenotype can be inferred from direct measurement and the pattern of mutations in resistant isolates. Both the clinical DAP-R strains *E. faecalis* and *E. faecium* displayed increased positive surface charge relative to their DAP-S counterparts (Arias et al., 2011; Mishra et al., 2012). Mutations impacting the *dlt* operon and *mprF* have been associated with DAP-R in *E. faecium* (Diaz et al., 2014). Further, *in vitro* adaptation of *E. faecium* to DAP led to the identification of additional genes associated with increases in cell surface charge, including *yvcRS*, *oatA*, and *divIVA* (Prater et al., 2019). Despite these observations, levels of lysyl-PG do not show a predictive trend in net surface charge in DAP-R strains (Mishra et al., 2012).

Alteration of membrane fluidity has also been observed in association with changes in DAP susceptibility. Differences in the length, saturation, and cyclization of fatty acyl chains of individual lipid species influence fluidity by altering lipid packing, and DAP has been reported to preferentially locate to regions of increased fluidity (Müller et al., 2016). Thus, changes in fluidity may alter DAP insertion and oligomerization in the membrane. Decreases in membrane fluidity (i.e., more rigid membranes) are associated with DAP-R resistance in enterococci. In *E. faecium*, this phenotype was associated with a decrease in the total proportion of unsaturated fatty acids and an increase in cyclic fatty acids which may be associated with mutations in *cfa* (cyclopropane fatty acid synthase) (Tran et al., 2013b; Diaz et al., 2014). Increased membrane rigidity has also been described in *E. faecalis* in association with changes in genes involved in the lipid metabolic pathway, including *cls, gdpD* (glycerophosphodiester phosphodiesterase), and *dak* (encoding a homologue of Fak, the staphylococcal fatty acid kinase) (Miller et al., 2019).

Further evidence supporting the potential importance of acyl-chain composition comes from a series of experiments examining the influence of exogenous fatty acids on the enterococcal membrane. Using laboratory isolates of *E. faecalis*, Harp and colleagues demonstrated that supplementation of growth media with oleic and linoleic acid induced tolerance to membrane stress, including protecting against DAP mediated killing (Saito et al., 2014; Harp et al., 2016). Subsequent studies profiling the changes in *E. faecalis* membranes associated with supplementation of a range of both saturated and unsaturated fatty acids found that exogenous fatty acids were rapidly incorporated into the acyl-chains of phospholipids. Unlike oleic acid, supplementation with saturated fatty acids and the native enterococcal monounsaturated fatty acid *cis*-vaccenic acid did not confer a survival advantage in the presence of DAP (Saito et al., 2017) while other combinations of “protective” fatty acids could induce DAP tolerance (Brewer et al., 2020). Thus, it is likely that variations in acyl-chains, as well as overall lipid species, play a role in protecting the membrane against daptomycin induced stress.

### Staphylococcus aureus

*S. aureus* is an important pathogen that causes a wide range of infections including cellulitis, bacteremia, and infective endocarditis (Tong et al., 2015). Vancomycin has been the mainstay of therapy for infections due to methicillin-resistant MRSA, however DAP has seen increasing use as salvage therapy for recalcitrant MRSA infections (Lee et al., 2018). While DAP retains activity against the vast majority of MRSA isolates, the overlap of genetic pathways leading to vancomycin-intermediate *S. aureus* (VISA) and heterogenous VISA isolates and decreased susceptibility to DAP has contributed to the emergence of DAP-R on therapy (Patel et al., 2006; Julian et al., 2007; Kelley et al., 2011).

Similar to enterococci, resistance to DAP has been linked to mutations in genes encoding TCS (*vraSR, yycFG*) and lipid biosynthetic enzymes (*pgsA, cls*, and *mprF*), with subsequent alteration of membrane composition and surface charge associated with reduced binding of DAP (Table 1) (Friedman et al., 2006; Muthaiyan et al., 2008; Mehta et al., 2012; Peleg et al., 2012; Bayer et al., 2013). It has been noted that DAP-R associated mutations in *yycG* (also known as WalK, a histidine kinase sensor involved in cell wall biosynthesis) could play a role in modulating fatty acid biosynthesis and potentially membrane fluidity as shown by the role of the YycFG homologue in *S. pneumoniae* (Mohedano et al., 2005). Interestingly, *S. aureus* has also been shown to exhibit “lipid shedding,” where DAP exposure triggered active release of membrane phospholipids that were able to inactivate DAP and protect from bacterial killing (Pader et al., 2016). This phenomenon has since been shown in other organisms, including in *E. faecalis* and *Streptococcus* spp. (Ledger et al., 2022).

Although a variety of lipids exist in staphylococcal membranes, the major phospholipids include PG, CL, and lysyl-PG, with PG and lysyl-PG being the most abundant (DeMars et al., 2020). Like enterococci, membranes from DAP-R *S. aureus* tend to have decreased amounts of PG, with increases in lysyl-PG which are closely correlated with mutations in *mprF*. Mutations in *mprF* are thought to lead to a gain-of-function, with increased production and/or flipping of lysyl-PG into the outer leaflet of the membrane (Ernst et al., 2009; Slavetinsky et al., 2022). In a DAP-S/DAP-R clinical strain pair, Jones et al. showed increased levels of lysyl-PG with decreased levels of PG in the resistant isolate (Jones et al., 2008). Other independent studies which analyzed MRSA strain pairs containing mutations in *mprF*, *yycG* and/or *cls2* showed similar increased levels of lysyl-PG in the DAP-R strains (Peleg et al., 2012; Mishra and Bayer, 2013). Conversely, a DAP-R MRSA strain containing mutations in *pgsA* (encoding PG synthase), *mprF*, and *yycG* amongst others showed reduced levels of PG, but also reduced levels of lysyl-PG and CL (Hines et al., 2017). This may be explained by the hypothesis that, in addition to electrostatic repulsion, MprF-mediated resistance may also reduce the available pool of PG for DAP to target, which has been supported through biochemical work using large unilamellar vesicles (Kilelee et al., 2010). Mutations in *dltABCD* (involved in D-alanylation of teichoic acids) have also been associated with DAP-R in *S. aureus* (Bayer et al., 2013; Bayer et al., 2016). Gain-of-function mutations in this operon are proposed to increase overall cell surface charge through alanylation of lipoteichoic acids and wall teichoic acids (Table 1; Figure 2), and deletion of *dlt* results in increased susceptibility to cationic antimicrobial peptides (Peschel et al., 1999).

Mutations in *cls2*, one of the two staphylococcal CL synthases, have been implicated in DAP-R. Alterations of the enzyme resulted in increased biosynthetic activity, increased levels of CL, decreased levels of PG, and no changes in lysyl-PG levels. CL-rich membranes have shown to have increased thickness by neutron reflectometry, which was associated with decrease in penetration and aggregation of DAP. This impaired ability of DAP insertion and translocation, which was postulated as the as mechanism underlying DAP-R in these strains (Jiang et al., 2019). Increases in CL and decreased levels of PG has also been linked to DAP-tolerance in serum-adapted strains of *S. aureus* (Ledger et al., 2022).

In addition, changes in membrane fluidity also correlate with decreased DAP bactericidal activity. In contrast to enterococci, clinical isolates of *S. aureus* have in general shown increases in membrane fluidity associated with DAP-R (Jones et al., 2008; Mishra et al., 2011a; Mishra and Bayer, 2013). While no significant differences in fatty acid content in saturation levels, length, or branching, the fluidity changes may be explained by membrane carotenoid content of *S. aureus*. In many clinical isolates, there was a statistically significant decrease in the membrane carotenoid staphyloxanthin accompanied by a decrease in membrane fluidity. Staphyloxanthin is expressed by the majority of *S. aureus* isolated from infections and has previously been implicated in protecting the bacteria from DAP and antimicrobial peptide mediated killing, although prior studies in laboratory strains associated resistance with increased staphyloxanthin content and more rigid membranes (Mishra et al., 2011b). Interestingly, a more rigid membrane phenotype has also been observed in isolates arising from *in vitro* adaptation experiments or exposure to other cell envelope active compounds (such as the lipoglycopeptide dalbavancin) for which there was also cross resistance to DAP (Mishra et al., 2009; Zhang et al., 2022). These changes were related to differences in the ratio of long and short-chain fatty acids; however, differences in strain background mutations and growth media prevent a direct comparison. It is not clear if the observed shifts in fluidity are mechanistically important in disrupting the binding or translocation of DAP in the membrane, or merely a consequence of the alterations of membrane composition.

### *Streptococcus* spp.

Viridans group streptococci (VGS) include a variety of species (i.e., *S. mitis, S. oralis, S. anginosus,* among others) that can cause severe infections including infective endocarditis, and resistance to commonly used antibiotics such as β-lactams is increasing (Doern et al., 1996; Marron et al., 2001; Prabhu et al., 2004). Unfortunately, high-level DAP-R [minimum inhibitory concentration (MIC) ≥ 256 μg/ml] can rapidly emerge in VGS upon DAP exposure (García-De-La-Mària et al., 2013; Akins et al., 2015).

While wild-type DAP-S cells contain PG and CL as the predominant phospholipids in the membrane, DAP-R VGS strains show no detectable PG or CL. Instead, cell membranes of resistant derivatives contain increased levels of the phospholipid precursor phosphatidic acid (PA) and decreased levels of phosphatidylcholine (not found in *E. faecalis* or *S. aureus*) (Adams et al., 2017; Mishra et al., 2017; Tran et al., 2019). This correlates with the identification of loss-of-function mutations in *cdsA*, a gene that encodes the phosphatidate cytidyltransferase enzyme (which generates the substrate CDP-diacylglycerol from PA for downstream phospholipid synthesis). The disappearance of CL and PG in various VGS strains harboring mutations in *cdsA* and/or *pgsA* was confirmed by lack of fluorescence in binding studies with 10-N-nonyl acridine orange NAO (Mishra et al., 2017), a fluorescent dye which binds APLs (Mileykovskaya and Dowhan, 2000).

Despite the lack of PG and CL in the membrane, several patterns of DAP binding have been observed in DAP-R streptococci (Figure 2). In *S. oralis* strains that developed DAP-R in association with mutations in *pgsA*, binding of DAP to the cell membrane appears to be uniform, but with significantly less binding overall (Tran et al., 2019). Conversely, strains that developed DAP-R via mutations in *cdsA* demonstrated hyperaccumulation of DAP in a small population of the cells (Mishra et al., 2017). The authors of this study hypothesized that a minority of bacterial cells may sequester DAP and allow the larger population to survive the antibiotic exposure, although the viability of cells as determined by propidium iodide uptake did not correlate with DAP hyperaccumulation. The discordance between a lack of PG and overall binding of DAP in VGS strains remains unexplained. While lack of PG and CL are consistent with many DAP-R VGS strains which have been adapted by serial passage or in an *ex vivo* simulated endocarditis vegetation model (SEV), there are other strains that showed no significant changes in lipid content (Kebriaei et al., 2019).

Overall, alterations in cell membrane fluidity have not been a consistent or predictable phenotype of DAP-R in VGS. Initial studies showed an increase in membrane fluidity was associated with resistance in *S. mitis/oralis* strains that were obtained from serial passage (Mishra et al., 2017; Mishra et al., 2020). These findings were later confirmed by Kebriaei et al. in an SEV model using the same strain. However, these changes may be isolate specific, as adaptation of a different strain background identified no differences in fluidity in the DAP-R derivative, and displayed a similar membrane lipid content relative to its DAP-S parent (Kebriaei et al., 2019). In a separate study, Tran et al. found no changes in fluidity between parental strains and evolved DAP-R derivatives of *S. mitis* or *S. oralis* strains despite the complete disappearance of PG and CL (Tran et al., 2019).

Similarly, alterations in cell surface charge have varied across DAP-R VGS. In *S. mitis* and *S. oralis* strains which developed DAP-R via mutations in *pgsA* or *cdsA*, Tran et al. found no changes in surface charge between parental strains and evolved DAP-R derivatives (Tran et al., 2019). In a serial passage experiment, DAP-R in *S. mitis/oralis* SF100 was associated with a decrease in net surface positive charge while maintaining the similar overall DAP binding profiles between the DAP-R and DAP-S strain (Mishra et al., 2020). However, in the SEV model Kebriaei et al. found no difference in surface charge in the DAP-R SF100 strain (Kebriaei et al., 2019). Furthermore, DAP-R in *S. anginosus* has been linked to changes in capsular polysaccharide and other cell surface modification genes that may affect surface charge. Substitutions in genes encoding *cls, yycG* and the *dlt* operon were identified in the DAP-R strain (Rahman et al., 2016) however, the exact contribution of surface charge to DAP-R in VGS remains unclear.

In summary, changes in lipid content associated with DAP-R in *S. mitis/oralis* seem to be dependent on strain and the type of mutation present despite a similar phenotype of high-level DAP-R (MIC ≥256 μg/ml). The precise mechanism of DAP-R in VGS or how this group of bacteria maintains cell envelope function and integrity despite the absence of PG and CL remains unclear. Further evaluation is warranted to determine the impact of membrane changes on DAP-R in VGS.

## Conclusion

The bacterial cell membrane is a dynamic structure that has evolved to adapt to changing environmental conditions. Understanding how bacteria alter their membrane in the face of external stress is critical to preserve the usefulness of membrane active antibiotics such as DAP. While the precise role of membrane lipid changes in the mechanism of resistance to DAP has yet to be completely explored, a deeper understanding of this process can be leveraged to overcome the limitations of current therapeutics.
